# Analytical Representations of Elastic Moduli Data With Simultaneous Dependence on Temperature and Porosity

**DOI:** 10.6028/jres.109.036

**Published:** 2004-10-01

**Authors:** R. G. Munro

**Affiliations:** National Institute of Standards and Technology, Gaithersburg, MD 20899

**Keywords:** analytical model, ceramics, elastic moduli, polycrystalline materials

## Abstract

An analytical model providing simultaneous, self-consistent representations of the temperature and porosity dependence of the elastic and bulk moduli of polycrystalline ceramics is applied to data compiled from the literature for 24 oxide ceramics.

## 1. Introduction

Elastic deformation is one of the most important considerations in structural applications of solid materials. Indeed, elastic properties are commonly required in computer aided design and manufacturing techniques to simulate a product’s behavior under variable conditions of stress and temperature. Under such conditions, it is desirable to have a means of estimating the value of a property continuously at any temperature or stress according to the local operating conditions. Tabulated data sets, however, are discrete and may be relatively sparse, particularly with respect to the dependence on microstructure. While interpolation techniques can be used with tabulated data if sufficiently extensive data tables are available, such approaches are relatively cumbersome. A more succinct and efficient approach is to use semiempirical analytical models that incorporate both material and environmental factors within the model.

An opportunity to construct an analytical representation of the elastic moduli data evolved recently from an extensive compilation (NISTIR 6853) of the elastic property data for polycrystalline oxide ceramics [1]. In that work, data were collected from the technical literature, either as reported in textual or tabular formats or as digitized from graphical formats. Special attention was given to the dependence of the moduli on both porosity and temperature.

In the present work, we report the construction of analytical representations of the elastic moduli data using a single model in which the effects of porosity (*ϕ*) and temperature (*T*) are treated simultaneously. Results for this model, applied to the data in NISTIR 6853 to the extent that sufficient data were available to evaluate the parameters in the model, are presented for 24 material specifications.

## 2. Model

To construct a suitable model, we proceed heuristically, beginning with the assumption that a separation of variables may be applied to the dependence of elastic moduli on temperature and porosity. For any modulus, *M*(*T,ϕ*), of a given material composition, it is assumed that *ϕ* and *T* may be taken as independent variables, and hence that we may consider

$$M(T,\phi )=M_{T}(T)M_{\phi }(\phi )$$ (1)

such that our task is to find suitable representations for *M*_T_(*T*) and *M_ϕ_* (*ϕ*).

### 2.1 Temperature Dependence

Empirically, the temperature dependence of Young’s elastic modulus for most ceramics is relatively simple, generally decreasing monotonically with increasing temperature. At very low temperature, the slope of the modulus with respect to temperature must approach zero. On the basis of lattice dynamics, Born and Huang [2] estimated that the elastic constants should vary as *T*^4^ at low temperature. Above room temperature, the moduli generally decrease linearly with increasing temperature. To describe the behavior from low to high temperature, Wachtman et al. [3] suggested the empirical relation

$$E_{W}(T)=E_{0}-bT\;\exp(-T_{0}/T)$$ (2)

in which *E*_0_ is Young’s modulus at absolute zero, and *b* and *T*_0_ are parameters to be determined numerically from the observed data. Anderson [4] later provided a justification of an expression of this form for the bulk modulus and noted that the elastic modulus would be approximately of the same form if the temperature dependence of Poisson’s ratio could be ignored.

Empirically, graphs of elastic moduli data vs temperature exhibit very little curvature except at very low temperature. This lack of curvature causes numerical fitting routines to be rather insensitive to the exponential factor in Eq. (2). Consequently, the uncertainty in the value of the parameter, *T*_0_, is unacceptably large for most of the data used in the present work. For the present purpose, therefore, it suffices to consider only the simplified linear model

$$M_{T}(T)=M_{T}(0)(1-a_{M}T)$$ (3)

with the parameters rewritten as *M*_T_(0) and *a*_M_ for each modulus *M*.

### 2.2 Porosity Dependence

The porosity dependence of the elastic properties of solids has been the subject of extensive investigation for decades. Numerous studies have examined the role of pores as the second component of two-phase solid media [5–10]. Those works generally involve an analysis of the strain field in the composite body under the application of an external stress. Alternatively, several studies [11–17] have observed that stress internally is transmitted only over the areas of contact between the constituent particles or grains. As the body is densified, the contact area increases while the porosity decreases. Consequently, the porosity dependence of the elastic moduli should be governed by the contact area. More recently, detailed analyses of the effects of pore size and pore shape have begun to be performed in finite element computer simulation calculations [18,19].

In addition to these microstructural modeling efforts, many semiempirical analytical models have been proposed [20–29] and applied [30–37] to represent the general trend of elastic moduli with porosity. Analytical models are of considerable interest because of their potential use as smoothing and interpolation functions. Since these models only relate bulk elastic properties to the mean porosity, they generally do not represent detailed microstructural effects arising from varying pore shape, anisotropy, or nonuniformity. Their importance rests in their capacity to provide highly effective descriptions of the trends of the mean properties and characteristics of porous media.

Empirically, a simple linear model [20] may be adequate at very small porosity, but for most brittle materials, the elastic moduli vary approximately exponentially [22] for porosity up to about 30 %. At higher porosity, the elastic moduli may deviate significantly from an exponential dependence [38]. Several models treat porous media as a special case of a two-phase medium in which the second phase consists of pores [36]. Those models often express the moduli of porous materials as ratios, *P*_1_(*ϕ*)/*P*_2_(*ϕ*), of polynomials (*P*_1_ and *P*_2_) in the volume fraction of porosity (*ϕ*). Budiansky’s self-consistent model [30] is of this type and results in a pair of coupled equations for the bulk and shear moduli. Those relations are explicitly linear in porosity and implicitly nonlinear through the self-consistent dependence on Poisson’s ratio, *v*, which is itself dependent on porosity.

At very high porosity, other issues must be considered in determining the influence of porosity on elastic moduli. It is self-evident that the volume fraction of porosity of a solid material must be less than one (*ϕ* < 1) because the condition *ϕ* = 1 corresponds to no material at all. As the limit *ϕ* = 1 is approached, the contiguity of the assemblage of components becomes an important issue since the integrity of an elastic medium is dependent on the transitivity of forces between adjacent material components. Indeed, in studies applying percolation theory, analyses of minimum solid areas of idealized stackings, and other models focused on the stacking of geometric shapes, there arises the possibility of a critical porosity, *ϕ*_c_, at which the moduli must vanish [11]. Such studies pertain to the very important issue of the validity of interpreting such an assembly of material components as an elastic continuum. Phani and Niyogi [26] suggested that if we are to allow for a vanishing modulus, then Young’s modulus, *E*, should be proportional to a power of (1 – *ϕ*/*ϕ*_c_).

In the present work, elasticity, as a bulk concept, is taken to mean *a priori* that the spatial connectivity is sufficient to allow the bulk material to sustain an applied stress. For any such material, without exception, the elastic modulus does not vanish.

Assuming material contiguity, Wagh et al. [27] considered a model in which the material was assumed to be composed of a network of material chains and interposed with channels of open pores. For a one dimensional system, they obtained the closed form expression

$$E=E_{0}{\left(1-\phi \right)}^{n}$$ (4)

where *E* is Young’s modulus, and *E*_o_ and *n* are adjustable parameters. They then used numerical solutions to verify that the same expression should be valid also for a three dimensional system. That conclusion was consistent with the results of Gibson and Ashby [37] who obtained Eq. (4) for the specific case of cellular ceramics, with *n* = 2 for open cell structures and *n* = 3 for closed cells.

Among these various models, it may be noted that the suitability of the various analytical forms is not sharply distinguished over the observed range of porosity for polycrystalline ceramics. No one model seems to have a stronger theoretical justification than the others, and the empirical fits to the data are not sharply different. Additionally, the general trends of the elastic moduli data vs porosity, for polycrystalline ceramics, do not seem to depend greatly on the nature of the porosity since results for specimens from multiple sources conform to a single trend line. Neglecting such details, it is possible to derive [39] a simple effective medium theory for the porosity dependence of bulk moduli. In this approach, the classical model of an ionic solid [40] was taken as an idealized, pore free, reference system. That choice had the particular virtue of providing a closed form expression for the bulk modulus. It was noted that the introduction of porosity into such a system must increase the molar volume of the material, *M*/*ρ*, where *M* is the molecular mass and *ρ* is the bulk density. As a result, the mean interaction potential at a site must be reduced because the mean interparticle distance is increased. To account for this relaxation in the model system, the length scale was formally renormalized. The renormalized system was then related to the porous physical system by imposing the consistency condition that the equilibrium volume of the renormalized system be equal to the sum of the volume at zero porosity and the pore volume. The result was the closed form expression

$$B=B_{0}{\left(1-\phi \right)}^{m}$$ (5)

In this model, the exponent, *m*, was determined by the effective attractive component of the interaction potential and can be different from the exponent, *n*, found in the similar expression, Eq. (4), for Young’s modulus.

### 2.3 The General Model

The elastic properties of polycrystalline ceramics usually are approximately isotropic because of the randomness of the grain orientations, even when the individual grains are anisotropic. An exception to this usual circumstance occurs for textured materials in which the microstructure has partially aligned grain orientations. In the present work, we consider only polycrystalline ceramics that may be treated as isotropic materials. For this case, the elastic properties are fully described by any two of the elastic moduli.

Upon viewing the dependence on temperature and porosity separately, we have seen that the temperature dependence may be represented effectively by Eq. (3). For the porosity dependence, there are several alternatives, but only two of the models, Eq. (4) for the elastic modulus and Eq. (5) for the bulk modulus, have been derived in closed form from theoretical models. Combining these models in the manner of Eq. (1), we obtain the general model describing the simultaneous dependence of *E* and *B* on the variables *T* and *ϕ*.

$$E(T,\phi )=E_{0}(1-\alpha T){\left(1-\phi \right)}^{n}$$ (6)

$$B(T,\phi )=B_{0}(1-bT){\left(1-\phi \right)}^{m}$$ (7)

## 3. Discussion

**Fig. 1:**
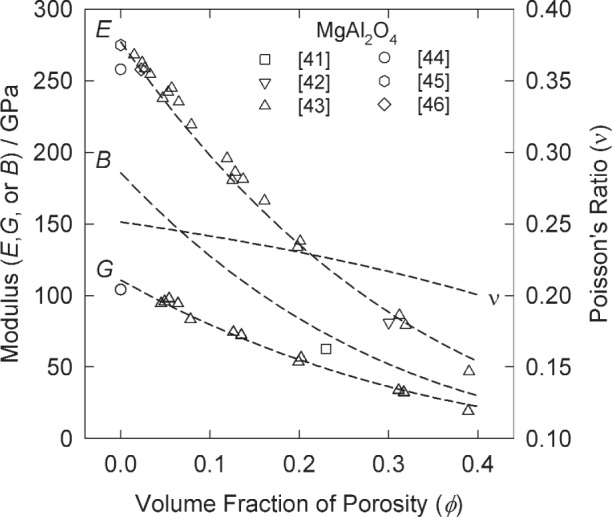
Elastic moduli (E, G, and B) and Poisson’s ratio (*v*) of MgAl_2_O_4_ vs. porosity, at room temperature. Numbers in square brackets, […], are references for the experimental data. The smooth curves comprise the fit of the model, Eqs. (6) and (7). (N.B.: The E value from [41] (square point at *ϕ* = 0.23) was treated as an outlier in fitting the E data.)

**Fig. 2:**
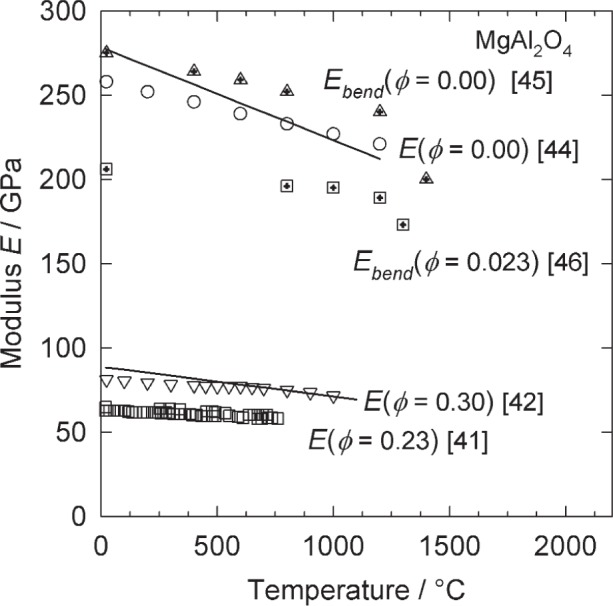
Elastic moduli of MgAl_2_O_4_ vs. temperature, for various values of the porosity. Numbers in square brackets, […], are references for the experimental data. The subscript, “bend,” indicates values derived from stress-strain bending curves; all other values were derived from ultrasonic measurement methods. The smooth curves comprise the fit of the model, Eqs. (6) and (7).

The model represented by Eqs. (6) and (7) has been applied to the data in NISTIR 6853, and the results are given in Table 1. An illustration of the typical fit of the model is given by the results for magnesium aluminate spinel [41–46], Fig. 1 and Fig. 2.

It should be noted that reports of elastic property data in the literature most commonly provide results for the elastic modulus and the shear modulus, *G*. The shear modulus for isotropic polycrystalline materials may be obtained from *E* and *B* as

$$G=\frac{3BE}{9B-E}$$ (8)

From this relation, it can be seen that *G* generally will not be of the same analytical form as *E* and *B*. For ceramics, the magnitude of *E* is typically on the order of twice that of *B*. Consequently, the relation in Eq. (8) can be expanded as

$$G=\frac{1}{3}E{\sum_{\varsigma =0}^{\infty }\left(\frac{E}{9B}\right)}^{\varsigma }$$ (9)

yielding

$$G\approx \frac{1}{3}E\left[1+\left(\frac{E}{9B}\right)+{\left(\frac{E}{9B}\right)}^{2}+\ldots \right]$$ (10)

from which it is seen that *G* may have a different functional dependence on *T* and *ϕ*, depending on the ratio (*E*/9*B*).

Similarly, we may note that Poisson’s ratio, *v*, is given by

$$v=\frac{1}{2}-\frac{E}{6B}$$ (11)

and depends directly on the ratio (*E*/6*B*). In the present work, the magnitudes of the products *aT* and *bT* in Eqs. (6) and (7) typically were found to have values of about 0.1 at 1000 °C. Hence, the ratio (*E*/*B*) is approximately

$$\frac{E}{B}\approx \frac{E_{0}}{B_{0}}\cdot (1-[a-b]T){\left(1-\phi \right)}^{n-m}$$ (12)

Consequently, Poisson’s ratio is not expected to be constant and may increase or decrease with temperature and porosity in a manner that reflects how the dependence of *E* differs from that of *B* with respect to the variables *T* and *ϕ*.

## 4. Conclusion

The condensation of a large tabulation of discrete data values into a representative analytical model is a data evaluation technique that optimizes the utility of the collected experiential data. The result is a succinct representation that enables the results to be more readily and consistently integrated into computerized design programs and enhances the use of the results in distributed data systems. The present work discusses the application of that technique to a compilation of elastic moduli data for a wide range of polycrystalline oxide ceramics. The model used in this work provides simultaneous, self-consistent representations of the elastic and bulk moduli for polycrystalline ceramics as functions of temperature and porosity.

## Figures and Tables

**Table 1:** Parameter values for the fit of the analytical model, Eqs. (6) and (7), for various oxide ceramics. The valid temperature and porosity ranges are indicated. The relative expanded uncertainties (coverage factor *k* = 2, 95 % confidence limit) for the computed elastic and bulk moduli were estimated as 5 %. Brackets, {}, indicate additional approximations were used, as indicated in the footnotes. *M*_r_ = molar mass. *ρ*_theo_ = theoretical mass density of the unstressed single crystal at room temperature

Material	*M*_r_ g mol^−1^	*ρ*_theo_ g cm^−3^	*T* range °C	Porosity range	*E*_0_ GPa	*a* 10^−4^ °C^−1^	*n*	*B*_0_ GPa	*b* 10^−4^ °C^−1^	*m*
Al_2_O_3_	101.961	3.984	0 to 1000	0 to 0.9	393	1.33	3.06	241	0.84	3.33
Al_6_Si_2_O_13_^a^	426.052	3.17	0 to 900	0 to 0.13	229	1.17	3.33	166	{1.16}	3.15
BeO	25.012	3.01	0 to 1400	0 to 0.16	386	0.77	1.96	350	1.18	1.61
Dy_2_O_3_^a^	372.998	8.161	0 to 900	0 to 0.2	186	1.37	3.81	144	{1.37}	3.52
Er_2_O_3_	382.516	8.651	0 to 1000	0 to 0.2	179	1.14	2.57	160	1.14	3.08
Gd_2_O_3_^d^	362.498	8.348	0 to 1400	0 to 0.37	157	1.46	2.32	114	1.47	2.19
HfO_2_(c,Pr)^a^^,^^e^	See^f^	n/a	0 to 1500	0 to 0.09	251	1.21	2.86	183	{1.21}	3.23
HfO_2_(c,Tb)^a^^,^^g^	See^h^	n/a	0 to 1650	0 to 0.18	229	1.41	1.78	186	{1.41}	2.78
HfO_2_(c,X)^b^^,^^i^	See^j^	n/a	0 to 1500	0 to 0.38	{256}	{1.52}	{3.01}	{200}	{1.70}	{4.09}
HfO_2_(PSH)^a^^,^^k^	See^l^	n/a	0 to 1600	0 to 0.12	{263}	{2.29}	{3.47}	{162}	{2.29}	{3.45}
Ho_2_O_3_	377.859	8.414	0 to 1000	0 to 0.18	175	1.08	2.60	155	0.98	3.43
Lu_2_O_3_	397.932	9.423	0 to 1000	0 to 0.34	204	1.03	3.12	161	0.24	4.27
MgAl_2_O_4_	142.266	3.572	0 to 1200	0 to 0.38	278	1.98	3.20	187	1.97	3.57
MgO	40.304	3.58	0 to 2500	0 to 0.26	310	1.63	3.81	164	1.23	2.64
Sc_2_O_3_	137.910	3.841	0 to 1400	0 to 0.3	229	1.22	2.97	148	0.98	2.45
Sm_2_O_3_	348.718	7.748	0 to 1300	0 to 0.38	150	2.00	2.85	125	1.73	3.45
ThO_2_^a^	264.037	10.0	0 to 1200	0 to 0.4	258	1.68	3.32	187	{1.66}	4.18
TiO_2_^c^	79.866	4.25	0 to 1600	0 to 0.35	286	1.52	4.99	{200}	{2.20}	{6.57}
Tm_2_O_3_	385.867	8.889	0 to 1000	0 to 0.24	185	0.88	3.07	147	1.63	2.18
YBa_2_Cu_3_O_6.9_	664.594	6.37	−268 to 25	0 to 0.5	150	1.54	3.70	69	1.84	3.19
Y_2_O_3_	225.810	5.03	0 to 1600	0 to 0.37	176	1.37	2.47	147	1.93	3.27
Yb_2_O_3_	394.078	9.2932	0 to 1000	0 to 0.27	199	0.90	2.61	155	1.24	2.83
ZrO_2_(m)^m^	123.223	5.6	0 to 1000	0 to 0.2	244	2.86	3.79	170	3.19	3.49
ZrO_2_(c)^b^^,^^n^	See^o^	n/a	0 to 1600	0 to 0.2	{227}	{1.50}	{2.59}	{183}	{1.48}	{4.31}

a Neither *B*(*T*) nor *G*(*T*) was known. Parameters were estimated using *a_G_* = *a_E_*.

b Parameters estimated using data from specimens with differing dopants.

c Optimization routine did not converge. Apparent midrange values were selected manually.

d Monoclinic structure.

e Cubic structure, HfO_2_·xPr_2_O_3_.

f Mr = 210.489 + 329.814x.

g Cubic structure, HfO_2_·xTb_2_O_3_.

h Mr = 210.489 + 365.849x.

i Cubic structure, HfO_2_·xX_2_O_3_, X = Er, Gd, Pr, Tb, and Y.

j Mr = 210.489 + xM_r_(X_2_O_3_).

k Partially stabilized hafnia, HfO_2_·xX_2_O_3_, X = Er, Eu, and Y.

l Mr = 210.489 + xM_r_(X_2_O_3_).

m Monoclinic structure.

n Cubic structure, ZrO_2_·xX_2_O_3_, X = Ca, Pr, Tb, and Y.

o Mr = 123.223 + xM_r_(X_2_O_3_).
